# Draft Genome Sequences of Two Veillonella tobetsuensis Clinical Isolates from Intraoperative Bronchial Fluids of Elderly Patients with Pulmonary Carcinoma

**DOI:** 10.1128/MRA.00397-19

**Published:** 2019-09-19

**Authors:** Izumi Mashima, Tohru Miyoshi-Akiyama, Junko Tomida, Ryo Kutsuna, Jumpei Washio, Nobuhiro Takahashi, Futoshi Nakazawa, Takuichi Sato, Yoshiaki Kawamura

**Affiliations:** a Department of Microbiology, School of Pharmacy, Aichi-Gakuin University, Nagoya, Aichi, Japan; b Pathogenic Microbe Laboratory, Research Institute, National Center for Global Health and Medicine, Shinjuku, Tokyo, Japan; c Division of Oral Ecology and Biochemistry, Tohoku University Graduate School of Dentistry, Sendai, Miyagi, Japan; d Health Sciences University of Hokkaido Graduate School of Dentistry, Ishikari-Tobetsu, Hokkaido, Japan; e Division of Clinical Chemistry, Department of Medical Technology, Niigata University Graduate School of Health Sciences, Niigata, Japan; University of Maryland School of Medicine

## Abstract

To date, Veillonella tobetsuensis has been known as an oral anaerobe and a facilitator of early-stage oral biofilm development with streptococci. Here, we report the draft genome sequences of 2 strains of V. tobetsuensis first isolated from intraoperative bronchial fluids of elderly patients with pulmonary carcinoma.

## ANNOUNCEMENT

Veillonella tobetsuensis is a strictly anaerobic Gram-negative coccus that was first isolated from a human tongue biofilm in 2013 (1). *V. tobetsuensis* has been isolated frequently from human oral cavities (2, 3), and its ability to form oral biofilms at the early stage with streptococci is significantly higher than that of other Veillonella species (4), suggesting that *V. tobetsuensis* has an important role in developing biofilms related to oral infectious diseases (4, 5).

Hasegawa et al. (6) isolated and identified *Veillonella* isolates as predominant anaerobes by 16S rRNA gene sequences from intraoperative bronchial fluids of patients with pulmonary carcinoma. However, 4 unknown isolates (PAGU 1578 to PAGU 1581) belonging to the genus *Veillonella* were isolated from two subjects (PAGU 1578 from a 70-year-old male and PAGU 1579 to PAGU 1581 from an 80-year-old female). These 4 unknown isolates were divided into two clones based on subject sources. Then, draft genome sequences of 2 representative unknown isolates, *Veillonella* sp. strains PAGU 1578 and PAGU 1579, were determined to identify them to the species level in this study.

The genomic DNA of these 2 isolates was extracted from 5-day cultures using phenol-chloroform extraction and ethanol precipitation (7). Further purification was carried out using the Wizard Genomic DNA purification kit (Promega) for high-throughput sequences. DNA libraries were prepared using the Nextera XT library kit (Illumina) according to the manufacturer’s instructions. DNA sequencing was performed at the National Center for Global Health and Medicine (Tokyo, Japan) using the HiSeq X Ten system (Illumina) with sequencing runs for paired-end sequencing, which achieved 150-bp read length and over 200-fold genome coverage. In total, 2,878,222 and 2,452,016 reads were obtained for PAGU 1578 and PAGU 1579, respectively, and they were checked for base quality (quality score limit, 0.05; ambiguous limit, 2; minimum number of nucleotides in reads, 15) and *de novo* assembled using CLC Genomics Workbench v. 11.01 (CLCbio). All final assemblies were annotated using the DDBJ Fast Annotation and Submission Tool (DFAST; https://dfast.nig.ac.jp/) with default parameters (8).

The draft genome sequences of PAGU 1578 and PAGU 1579 were assembled into 59 (*N*_50_ = 96,198) and 129 (*N*_50_ = 39,212) contigs and were 1,935,552 bp and 2,151,918 bp in size, respectively. These two draft genomes contained G+C contents of 38.4% and 38.5% and 1,789 and 1,869 coding sequences, respectively.

To investigate genomic comparison among closely related isolates of the genus *Veillonella*, average nucleotide identity (ANI) values were calculated using GENETYX v. 14 software (Tokyo Genetyx Co.) with default parameters. The results of BLAST calculation of ANI values between PAGU 1578 and PAGU 1579 or these two clinical isolates and *V. tobetsuensis* ATCC BAA-2400^T^, which is the most closely related species, were 96.73% and 96.16% to 97.05%, respectively. These results suggested that PAGU 1578 and PAGU 1579 were the same species, *V. tobetsuensis*.

This is the first report of *V. tobetsuensis* isolated from bronchial fluids, and their draft genome sequences are announced. Streptococci were also isolated from the same clinical samples as predominant bacteria with *Veillonella* species (6), suggesting that *V. tobetsuensis* might have the ability to facilitate biofilm infection in bronchial diseases, as suggested in oral infectious diseases (4, 5).

### Data availability.

These draft genome sequences of *V. tobetsuensis* PAGU 1578 and PAGU 1579 and the raw sequence data sets have been deposited at DDBJ/EMBL/GenBank under the accession numbers BJCQ01000001 to BJCQ01000133, BJCR01000001 to BJCR01000129, and DRA008107, respectively. The versions described in this paper are the first versions.

## References

[1] Mashima I, Kamaguchi A, Miyakawa H, Nakazawa F 2013 *Veillonella tobetsuensis* sp. nov., an anaerobic Gram-negative coccus isolated from human tongue biofilms. Int J Syst Evol Microbiol 63:1443–1449. doi:10.1099/ijs.0.042515-0. 22843723

[2] Mashima I, Nakazawa F 2013 Identification of *Veillonella tobetsuensis* in tongue biofilm by using species-specific primer pair. Anaerobe 22:77–81. doi:10.1016/j.anaerobe.2013.04.015. 23664905

[3] Theodorea CF, Mashima I, Thaweboon B, Thaweboon S, Nakazawa F 2017 Molecular detection of oral *Veillonella* species in the saliva of children with different oral hygiene status. Int J Curr Microbiol App Sci 6:449–461. doi:10.20546/ijcmas.2017.607.054.

[4] Mashima I, Nakazawa F 2014 The influence of oral *Veillonella* species on biofilms formed by *Streptococcus* species. Anaerobe 28:54–61. doi:10.1016/j.anaerobe.2014.05.003. 24862495

[5] Mashima I, Nakazawa F 2017 Role of autoinducer-2-like molecule from *Veillonella tobetsuensis* in *Streptococcus gordonii* biofilm formation. J Oral Biosci 59:152–156. doi:10.1016/j.job.2017.06.002.

[6] Hasegawa A, Sato T, Hoshikawa Y, Ishida N, Tanda N, Kawamura Y, Kondo T, Takahashi N 2014 Detection and identification of oral anaerobes in intraoperative bronchial fluids of patients with pulmonary carcinoma. Microbiol Immunol 58:375–381. doi:10.1111/1348-0421.12157. 24818822

[7] Marmur J 1961 A procedure for the isolation of deoxyribonucleic acid from micro-organisms. J Mol Biol 3:208–218. doi:10.1016/S0022-2836(61)80047-8.

[8] Tanizawa Y, Fujisawa T, Nakamura Y 2018 DFAST: a flexible prokaryotic genome annotation pipeline for faster genome publication. Bioinformatics 34:1037–1039. doi:10.1093/bioinformatics/btx713. 29106469 PMC5860143

